# Supplementary material for: Perceptions of Harmfulness of Heated Tobacco Products Compared to Combustible Cigarettes among Adult Smokers in Japan: Findings from the 2018 ITC Japan Survey
Source: Int J Environ Res Public Health. 2020 Apr 1;17(7):2394. doi: 10.3390/ijerph17072394 (PMC7177718; doi:10.3390/ijerph17072394)
Supplement: Supplementary file 1 [file ijerph-17-02394-s001.zip › ijerph-755134 R1 supplementary tables Final.docx]

**Table S1.** Relative Perceptions of Harmfulness of HTPs Compared to Cigarettes Among Smokers in Japan in 2018.

|  | **Overall ***  ***N* = 3600** | **Exclusive Smokers ***  ***n* = 2614** | **Concurrent Users ^†^**  ***n* = 986** | **Odds Ratio (95% CI)** |
| --- | --- | --- | --- | --- |
|  | **Weighted % (95% CI)** | | |  |
| HTPs are Less Harmful (*n* = 1723) | 47.5% (45.6–49.5%) | 43.8% (41.4–46.2%) | 62.1% (58.0–66.1%) | Reference |
| HTPs are Equally as Harmful (*n* = 867) | 24.6% (22.9–26.2%) | 24.9% (23.1–26.8%) | 22.1% (18.8–25.5%) | 1.60 (1.3–2.0) *** |
| HTPs are More Harmful (*n* = 87) | 1.8% (1.4–2.3%) | 1.9% (1.4–2.4%) | 1.5% (0.7–2.4%) | 1.73 (1.00–3.1) |
| I Don’t Know (*n* = 923) | 26.1% (24.4–27.8%) | 29.4% (27.4–31.4%) | 14.3% (11.4–17.1%) | 2.9 (2.3– 3.8) *** |

Data are weighted and adjusted. HTP: Heated tobacco products. CI: Confidence interval. * Model 1: Covariates: age, gender, education, income, smoking frequency (daily vs. non-daily), current HTP use (daily vs. non-daily vs. no current use/never used). Wald: 48.91, *p* < 0.0001. ^†^ Model 2: Tested perceptions of harmfulness between users groups (exclusive smokers vs. concurrent users). Covariates included: age, gender, education, income, and smoking frequency (daily vs. non-daily). Wald: 54.24, *p* < 0.0001. Main effect for user group = *p* < 0.0001. **^±^** test between user groups.*** *p* < 0.0001.

**Table S2.** Relative Perceptions of Harmfulness of HTPs Compared to Cigarettes Among Concurrent Users Stratified by HTP Use Frequency (Frequent HTP Users vs. Infrequent HTP Users).

|  | **Frequent HTP Users**  ***n* = 548 (55.6%)** | **Infrequent HTP Users**  ***n* = 438 (44.4%)** | **Odds Ratio (95% CI)** |
| --- | --- | --- | --- |
|  | **Weighted % (95% CI)** | |  |
| HTPs are Less Harmful (*n* = 646) | 71.7% (67.0–76.1%) | 57.1% (51.9–62.2%) | Reference |
| HTPs are More/Equally as Harmful (*n* = 222) | 18.6% (14.7–22.6%) | 28.5% (23.9–33.2%) | 0.5 (0.4–0.7) |
| I Don’t Know (*n* = 118) | 9.0% (5.9–12.1%) | 13.6% (10.2–17.1%) | 0.5 (0.3–0.8) |

Data are weighted and adjusted. HTP: Heated tobacco products. *n* = 1001; Wald: 2.13, *p* = 0.001. Main effect of HTP use frequency: *p* = 0.0002. Model adjusted for: age, gender, education, income, and smoking frequency (daily vs., non-daily).

**Table 3.** Relative Perceptions of Harmfulness of HTPs Compared to Cigarettes Among Concurrent Users Stratified by Smoking and HTP Use Frequency: Predominant Smokers Vs. Concurrent Daily Users vs. Concurrent Non-daily Users.

|  | **Predominant Smokers (A)**  ***n* = 528 (53.8%)** | **Concurrent Daily Users (B)**  ***n* = 396 (40.3%)** | **Concurrent Non-daily Users (C)**  ***n* = 58 (5.9%)** | **Odds Ratio (95% CI)** |
| --- | --- | --- | --- | --- |
|  | **Weighted % (95% CI)** | | |  |
| HTPs are Less Harmful (*n* = 644) | 58.8% (53.9%–63.6%) | 73.8% (68.4%–79.1%) | 56.4% (40.9%–70.9%) | A-B: 1.9 (1.4 –2.7)  B-C: 2.1 (1.1–4.2) |
|  |  |  |  | A-C: 0.9 (0.5–1.8) |
| HTPs are More/Equally as Harmful/ I Don’t Know (*n* = 338) | 41.2% (36.4%–46.2%) | 26.8% (21.9%–32.5%) | 43.6% (29.2%–59.2%) | Reference |

Data are weighted and adjusted. HTPs: Heated tobacco products. Predominant HTP users were excluded due to low sample size (*n* = 4). *n* = 982; Wald: 2.47, *p* = 0.003; Main effect of User Group: *p* = 0.0005.
