# Supplementary figures and images for: Perceptions of Harmfulness of Heated Tobacco Products Compared to Combustible Cigarettes among Adult Smokers in Japan: Findings from the 2018 ITC Japan Survey
Source: Int J Environ Res Public Health. 2020 Apr 1;17(7):2394. doi: 10.3390/ijerph17072394 (PMC7177718; doi:10.3390/ijerph17072394)

**Figure S1.** Study Flow Diagram

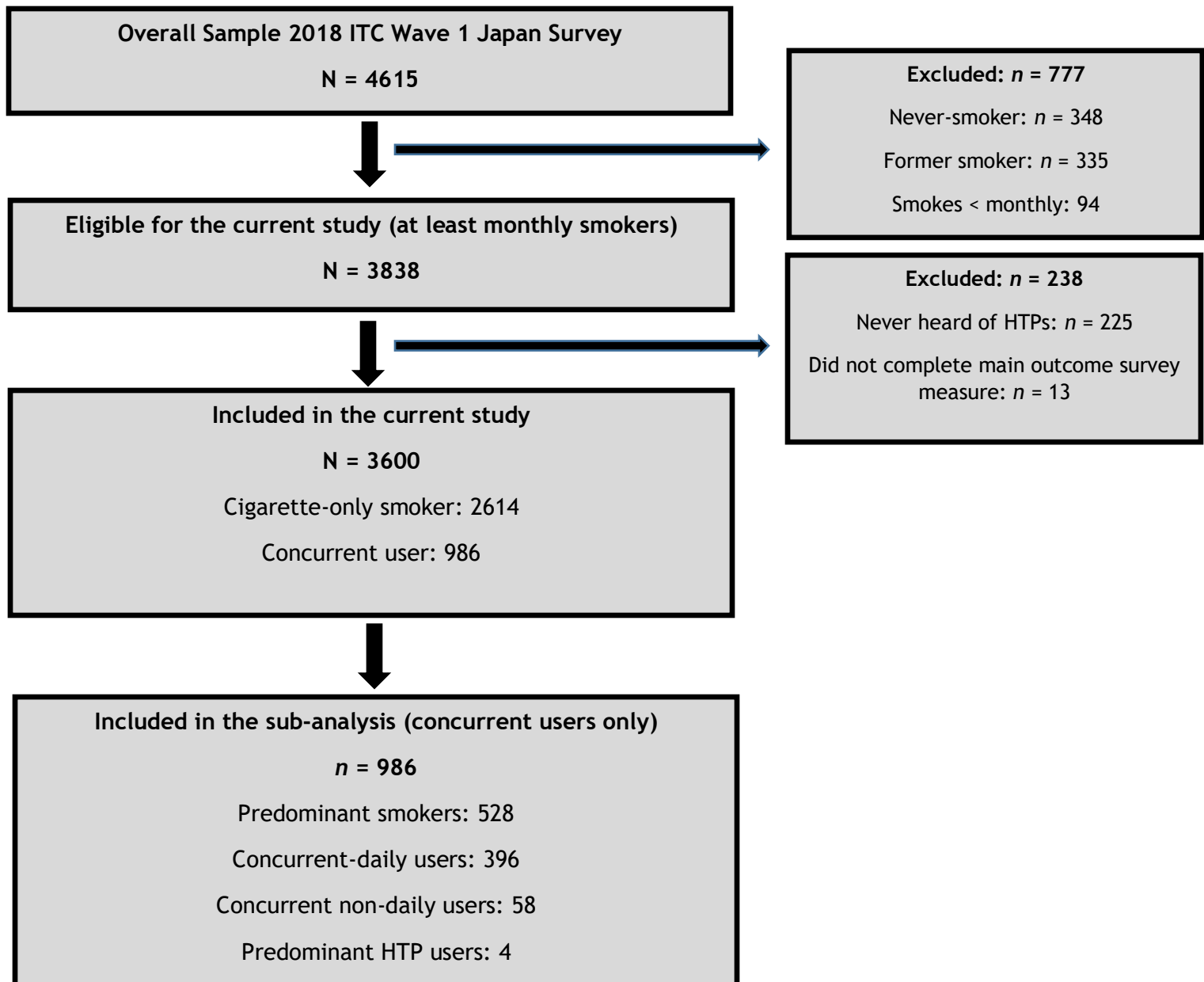

Supplement: Supplementary file 1 [file ijerph-17-02394-s001.zip › ijerph-755134 Figure S1 author proofreading.pdf]
